# People behavioral during health information searching in COVID-19 era: a review

**DOI:** 10.3389/fpubh.2023.1166639

**Published:** 2023-08-10

**Authors:** Haitham Alzghaibi

**Affiliations:** Department of Health Informatics, College of Public Health and Health Informatics, Qassim University, Albukayriah, Saudi Arabia

**Keywords:** health information seeking, health behaviors, Internet, COVID-19, patients behavior

## Abstract

**Background:**

The COVID-19 pandemic has led to an increase in health information-seeking behavior (HISB) on the Internet.

**Objective:**

This review aims to identify and synthesize the available evidence on health information-seeking behavior on the Internet during and after the COVID-19 pandemic.

**Methods:**

Electronic search of databases was conducted on PubMed, ScienceDirect, Scopus, and Taylor and Francis Online to identify relevant articles. Studies that examined health information-seeking behavior on the Internet during or after the COVID-19 pandemic were included. Data from the included studies were subjected to a thematic analysis.

**Results:**

A total of 355 articles were identified in the initial database search. After screening, 15 articles were included in this review paper, with a population of 33,326. Search engines, social media, and news portals were the most commonly used information sources. The primary motivators for seeking health information online were curiosity, catching up with updated information, and paying attention to the COVID-19 transmission. Participants’ satisfaction with the information obtained online was positive in most studies. The online query for all items related to COVID-19 and health increased during the pandemic. The most searched topics were symptoms of COVID-19, restrictions, current prevalence/spread of COVID-19, and preventive measures. Higher scores in digital health literacy (DHL) were associated with a well-established and effective health information-seeking behavior.

**Conclusion:**

The findings of this review provide insight into the patterns and trends of health information-seeking behavior on the Internet during and after the COVID-19 pandemic. The results suggest that search engines, social media, and news portals remain key sources of information during the pandemic. It also assessed the relationship between the DHL and the HISB and found that having a good DHL generally meant a good HISB.

## Introduction

Health information seeking (HIS), also referred to as health information seeking behavior (HISB), is the process of looking for and obtaining information related to health or healthcare (1). It can include searching for information about specific illnesses, treatments, or medications (2–4), as well as seeking general information about health and wellness (3). Information seeking can be done for various reasons, such as to better understand a person’s or family member’s health condition, make informed healthcare decisions, or improve overall health and wellness (2, 5). Health information seeking can be done through various online and offline sources, including social media, television and radio programs, scientific papers, books, online health information websites, healthcare professionals, family, and friends (6). In the case of a pandemic, getting medical assistance and obtaining relevant healthcare information becomes a challenge. Specifically, during the COVID-19 pandemic, access to health information became more and more limited to the Internet and telecommunication. The implementation of measures such as movement restrictions and social distancing, which were introduced as a response to the COVID-19 pandemic, led to this outcome. According to a study by the Pew Research Center, in August 2021, about four in 10 adults in the U.S. got information about the COVID-19 from social media platforms (7). In another report based on Google trends for 2020 and published by Forbes, 45% of the search queries from the U.S. were “Coronavirus,” and 35% of the terms were “Covid” (8). The use of the Internet for health information-seeking has also been well documented in research literature. A review of 37 studies found the Internet to be the most used source of health information, with approximately 80% of Internet users seeking health information online (9). Also, in a study published by Bujnowska-Fedak et al. (10), 76.9% of the participants used the Internet as a source of health information. The studies by Mitchell and Liedke (7) and Chamary (8) reported an increase in the use of the Internet for searching for information during the COVID-19 pandemic period as compared to the period before. This increase was primarily associated with the spread of the coronavirus and the restrictions or changes that the COVID-19 pandemic brought about (8, 9). From the previous statement, it may be correct to say that the use of the Internet for HIS went down after the pandemic since it was the pandemic that brought it up. However, rather than make assumptions, it is prudent to use published research material to explore and understand the HISB on the Internet even after the COVID-19. This review thus aims to understand the health information-seeking behavior on the Internet during and after the COVID-19 pandemic.

## Methods

### Guideline

This review is reported following the guidelines published in the Preferred Reporting Items for review and Meta-Analyses (PRISMA) statement (11).

### PICO statement

The following statements were used to help define the research question and develop a clear and focused search strategy.

P: Adult population (18 years or older).

I: Health information-seeking behavior on the Internet.

C: No specific intervention or comparison group.

O: Patterns and trends, factors influencing credibility, or other outcomes related to health information-seeking behavior during and after the COVID-19 pandemic.

### Search strategy

A systematic search of academic databases for articles was done on PubMed, ScienceDirect, Scopus, and Taylor and Francis Online. A search string was developed from the topic keyboards and used in searching the index databases. The search string used was (“health information seeking behavior” OR HISB OR “health information seeking”) AND (Internet OR online OR “social media” OR website) AND (COVID-19 OR corona).” It was used in all databases with no filter applied.

#### Research question

The research question for this review is: What are the pattern and factors of health information-seeking behavior on the Internet during and after the COVID-19 and relationship between DHL and HISB.

### Inclusion and exclusion criteria

#### Inclusion criteria

- Studies that examine health information-seeking behavior on the Internet in adults (18 years or older) during or after the COVID-19 pandemic.
- Studies that focus on the patterns and trends, information sources, motivators, satisfaction and evaluation of the information, and the relationship between DHL and HISB.
- Studies that use quantitative, qualitative or mixed-methods research designs.
- Studies published in English language and published during or after 2020.
- Studies published in peer-reviewed journals.

#### Exclusion criteria

- Studies that focus on health information-seeking behavior in children or adolescents.
- Studies that focus on health information-seeking behavior related to diseases other than COVID-19.
- Studies that focus on health information-seeking behavior related to mental health or well-being only.
- Studies that focus on health information-seeking behavior in healthcare professionals or students.
- Studies that use non-research methods, such as editorials, letters, commentaries, or conference abstracts.
- Studies not published in English

### Quality assessment

The Newcastle-Ottawa Scale (NOS) criteria were utilized, which has 10 points distributed across three domains (selection, comparability, and outcome) for observational studies (12). We included only those studies that scored ≥5 points on the modified NOS components.

### Data synthesis

The data from the included studies were synthesized using a narrative synthesis approach. A descriptive synthesis was conducted for the quantitative studies, while a thematic synthesis was conducted for the qualitative studies. The data from the included studies were organized into themes and sub-themes based on the patterns and trends, information sources, motivators, satisfaction and evaluation of the information, and the relationship between digital health literacy (DHL) and health information-seeking behavior.

### Study selection

**Figure 1:**
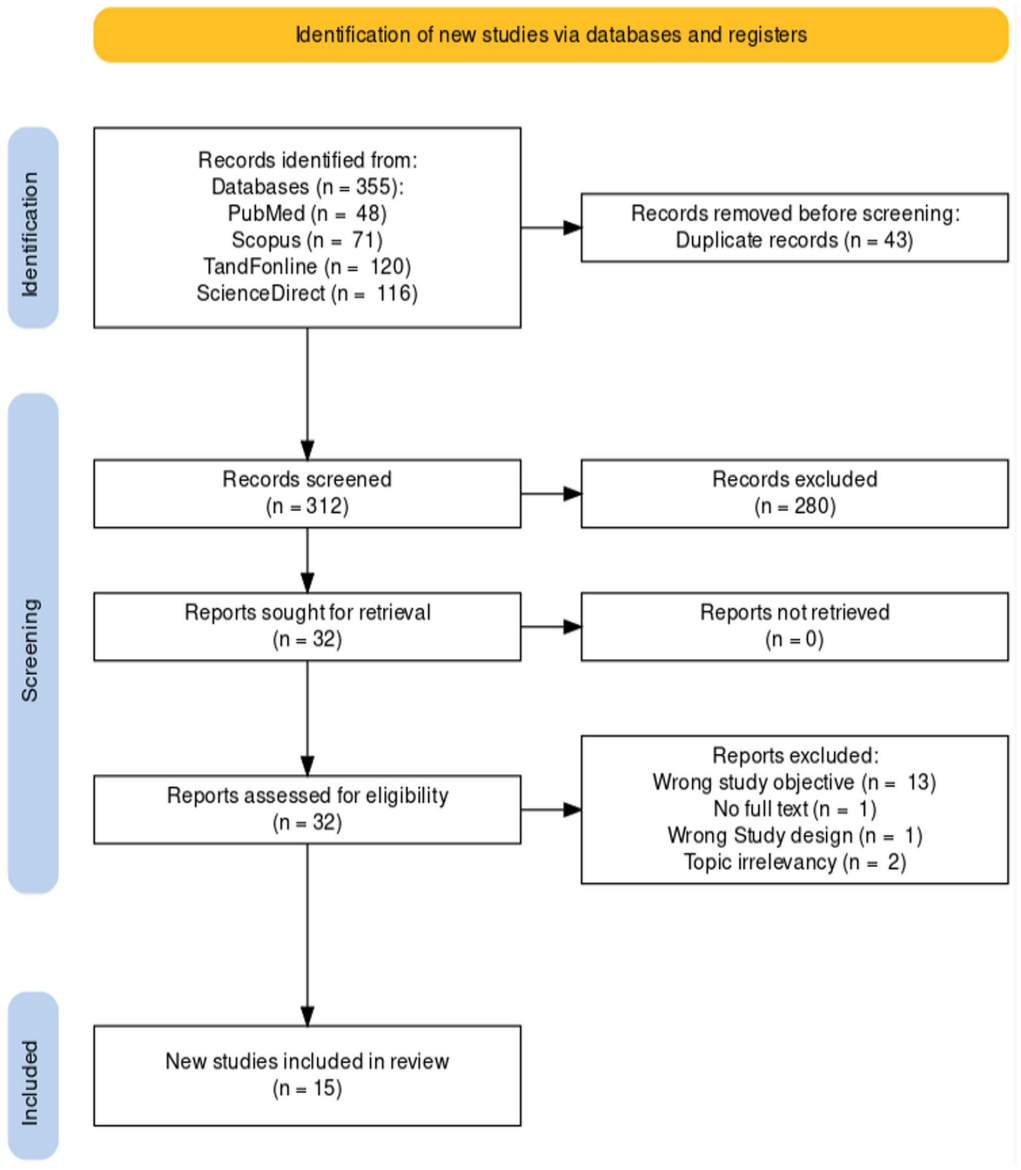
PRISMA diagram showing the study selection process.

A total of 355 articles were found in the initial database search, out of which 43 duplicates were removed. Following the title and abstract screening, 280 articles were excluded due to being irrelevant to the topic and not meeting the inclusion criteria. After a full-text reading of the remaining 32 articles, 13 were excluded for having wrong study objectives, one for lack of full-text material, one for wrong study design, and two for being irrelevant to the topic. Eventually, only 15 articles were deemed eligible for inclusion in this review paper. The selection process is illustrated in Figure 1.

### Data extraction

The methodology of this study involved an integrative review approach, which allowed for the integration of various research designs, including quantitative, qualitative, and mixed-method designs. To synthesize the integrative data, qualitative techniques were utilized, which enabled iterative comparisons across primary data sources. This study reviewed 15 articles and thoroughly analyzed their abstracts, results, and discussions to identify data that could answer the research questions. The data was then extracted into a predefined spreadsheet table. These attributes are author name, publication year, study type/design, data source, study region, participant size, and participant type. Thematic analysis was conducted, which involved identifying themes and sub-themes by observing patterns and clustering and counting them while noting similarities and relationships within the data. This rigorous approach allowed for a comprehensive analysis of the literature related to health information-seeking behavior on the Internet during and after the COVID-19.

## Results

### Characteristics of included studies: summary

This paper included 14 cross-sectional studies and one longitudinal study with a total S. R (see Table 1) population of 33,326 people. This number is for all included studies except four. These four studies are Mangono et al. (18), Rew et al. (20), and Rovetta and Bhagavathula (23), which all used RSV data from Google Trends, and either Zhao and Basnyat (27) and Zhao et al. (28), which used the same study sample. There were variations in the data collection method, study area, and participant type across included studies. Even though all of the studies looked at HISB during the COVID-19 pandemic, none of the studies looked at how the HISB had changed past COVID-19. This made it impossible for a during-and-after comparison to be made, as was intended in this review. However, some studies (18–20, 23) provided a brief overview of how some of the aspects of HISB, e.g., the use of certain keywords, had changed from before the pandemic to during the pandemic.

**Table 1:** Study descriptor table.

References	Study design	Data source	Area	Participants size	Participants type
Abdoh (13)	CSS	Semi-structured phone interviews and online survey	Saudi Arabia	319 (48.0 % female)	University students
Bak et al. (14)	CSS	Survey questionnaire	Denmark	1,518 (83.4% female)	University students
Dadaczynski et al. (15)	CSS	Survey questionnaire	Germany	14,916	University students
Hsu (16)	CSS	Survey questionnaire	Taiwan	101	University students
Htay et al. (17)	Web-based CSS study	Survey questionnaire	China, Malaysia, and the Philippines.	5,302 (75% were female)	University students
Mangono et al. (18)	LS	Google trends data	United States	N/A	Internet users
Neely et al. (19)	CSS	Web-based survey	United States	1,003 (51.2%)	Social networking site users
Rew et al. (20)	CSS	Google trends data	India	N/A	General population
Rosário et al. (21)	CSS	COVID-HL survey data	Portugal	3.084 students (75.7% women)	University students
Rovetta and Bhagavathula (22)	CSS	Google trends data	Italy	N/A	N/A
Vrdelja et al. (23)	CSS	Survey questionnaire	Slovenia	3,621 (70% female)	University students
Zakar et al. (24)	CSS	Web-based interviews	Pakistan	1,747 (52.7% female)	University students
Zhang et al. (25)	CSS	Questionnaires	China	219 (61.2% female)	Social media users
Zhao and Basnyat (26)	CSS	Data from Weibo	China	1,496	General public
Zhao et al. (27)	CSS	Data from Weibo	China	1,496	General public

CSS, cross-sectional study; LS, Longitudinal study.

All of the included studies were subjected to a quality appraisal process by following the NOS criteria, and all of them scored well in overall quality. None of the studies were low-quality studies, and hence no study was eliminated on the basis of methodological quality. The detail presentation of quality assessment for each study is presented in Table 1.

### Results of included studies: thematic analysis

#### Search strategy

Different techniques were used when searching for health information online, and variations existed in the terms used, frequency of use, and time spent searching. In Hsu (16) the study revealed that 59% of the participants used nouns, 43% used nouns, adjectives, and adverbs, and 28% used sentences as their keywords. Only 27% of participants used Boolean logic, while 12% limited the type of data searched and a mere 2% limited the date and language of data retrieved, indicating low usage of filtering methods to refine search results. The words used also varied across studies (22, 27). In Rovetta and Bhagavathula (22), where the search was for general health information, the most used search words were “coronavirus,” “novel coronavirus,” and “China coronavirus.” In Zhao et al. (27), where the search was for help and support for family members, the most used family words were “mum,” “dad,” and “elder at home.” These words were accompanied by a description of the health condition like hypertension, “diabetes,” and “heart disease,” (27).

To maximize search outputs, 27% of the participants in Hsu (16) used Boolean operators “AND,” “OR,” and “NOT.” 12% of them limited the type of data searched, and 2% specified a date range and language of preference (16). In Zakar et al. (24), 63.7% of the participants used English as their language during online research. 19.3% of the respondents used English plus the Urdu languages, and 17% of them used Urdu only.

Regarding time spent looking for information, the total browsing time when using webpages was an average of 5.54 min and the average time on each site was 2.39 min (16). In regards to frequency of seeking, Neely et al. (19) reported that at least 32.2% of the study population searched for information every day, 27% looked for information a few days per week, and 11.2% of the study population looked for information at least once a week (Table 2).

**Table 2:** Information sources.

Study	Information sources											
	search engines (e.g., Google, Bing, or Yahoo)	Social media (Facebook, Instagram, Twitter)	Wikipedia and other online-encyclopedias	YouTube	Blogs on health topics	Websites of *public* bodies (MOH, provincial health departments)	Websites of doctors/pharmaceutical companies/or health insurance companies	Guidebook/Support communities/online communities/chat rooms	News portal (e.g., newspapers, T.V. stations)	Health portals	Magazines/Periodicals	Online consultation
Bak et al., (14)	77%	52%	19%	21%		78%	32%	29%	87%			
Dadaczynski et al. (15)	84.40%	37.70%	31.40%	38.40%	11.60%	55.70%	25.30%	4.60%	83.70%	12.90%		
Hsu (16)					22%	45%		13%	40%			
Htay et al. (17)	92%	88.40%	55.30%	70.60%	46.60%	68.20%	35.30%	38.8 %	82%	47.40%		
Neely et al. (19)		76%										
Vrdelja et al. (23)	84.80%				9.40%	69.70%	14.20%		66.90%			6%
Zakar et al. (24)	43.80%	39.90%	26.10%	39.70%	29.40%	26.50%			36.70%			
Zhang et al. (25)		82.65%										

### Information sources

Most of the study participants used search engines (15, 17, 23, 24), and social media (19, 25). In Bak et al. (14), the most used information source was news portals at 87%, followed by websites of public institutions like the ministry of health or provincial health departments at 78%. In Abdoh (13), where source use was not reported in percentages, search engines were the most utilized, followed by social media and then YouTube. Wikipedia and other online encyclopedias were the least used information sources (13).

#### Gender on information sources

From reports, gender seemed to affect the frequency of seeking information (27), and the source used (15). In Dadaczynski et al. (15), female students used social media and health portals more frequently than males. The males preferred using Wikipedia and YouTube. Zhao et al. (27) assessed 2,405 unique users on Weibo in China and found that 69% were female. Also, in the particular context of the study, females posted two times the number that males posted (27).

#### A special look at social media use

Neely et al. (19) looked at the social media pages/accounts that their participants were following. 76.2% reported following at least one authoritative scientific source on social media during the pandemic. 27.6% followed the Centers for Disease Control, 26.9% followed their state public health department, and 26.4% followed their local public health department. 22.4% followed a known infectious disease expert, and 20.2% followed a physician of their liking.

### Factors in information-seeking behavior

#### Factor: information evaluation

Information evaluation is the process of assessing the quality, relevance, and reliability of information (10). This can involve evaluating the credibility of the information source and assessing the appropriateness of the information for the intended purpose (10).

Vrdelja et al. (23) and Zakar et al. (24) presented different aspects that the respondents claimed to look for when evaluating the credibility of a source. In Zakar et al. (24), 68.7% of the respondents required that information be up-to-date, 62.6% required that it be verified and 62.4% required that the information come from an official source. Also, in Vrdelja et al. (23), 99.3% required the information to be verified for it to be credible. However, in the same study, 18% of respondents did not mind the existence of conflicting information.

Despite these requirements for verification (23, 24), the question of whether they did practice evaluation or not still remained. In Hsu (16), only 36.4% of the respondents had sought a doctor’s professional view on information they saw on social media, 68.4% had discussed it with friends, and 57.6% had conducted a personal Internet search to verify the information.

#### Factor: information satisfaction

Information satisfaction is a measure of the effectiveness of the HISB process, which is defined by the amount of effort required to find sought information and the speed at which the information was obtained. 61% of the respondents in Htay et al. (17) reported information satisfaction. In Vrdelja et al. (23), 86.9% of the participants reported being at least satisfied with the online health information. This percentage was 85.8% in Zakar et al. (24) and 70.8% in Bak et al. (14).

Some of the predictors for positive information satisfaction were good DHL in information searching & evaluating reliability subscales, online privacy, and quality of Internet search strategy (17).

Regarding confidence in information on COVID-19 in social media, the reported confidence rates were 32.2% for Neely et al. (19).

### Trends in information-seeking behavior

Using relative search values (RSVs) presented by Google Trends, Mangono et al. (18) noticed that search rates for certain keywords and topics seemed to increase in anticipation of government announcements. For example, inquiries for social distancing increased, while inquiries for a bar/restaurant nearby decreased; this behavior was observed 3 days before WHO declared COVID-19 spread to be a pandemic and 8 days before the U.S. government released its official social distancing guidelines (18). Also, the search for “how to make a mask” had increased 12 days before the US Centers for Disease Control and Prevention issued a statement promoting the use of masks for the general public (18). Mangono et al. (18) also reported an increase in search for COVID-19 related news, care-seeking for COVID-19, fake news and coronavirus hoaxes (surged 38 times), social distancing and how to make masks, online shopping, and COVID-19–specific stimulus packages as well unemployment benefits. A decline was noted in the search for general health, i.e., urgent care and doctor appointments and health programs (i.e., health insurance, Medicaid, and Medicare) (18).

Rovetta and Bhagavathula (22) reported that in the early periods of the COVID-19 outbreak in Italy, there was a spike in searches regarding symptoms, face masks, and disinfectants. Later on, as the pandemic progressed, a huge spike in the search for the symptoms of COVID-19 was observed.

A change was also noticed in the social media space. In Neely et al. (19), 76.2% of its respondents had intentionally expanded their social media networks during the pandemic to include credible institutions, individuals, and sources.

The existence of COVID-19 seemed to affect not only the HISB for the COVID-19 virus alone but also other conditions. For example, the search for information on comorbid conditions like diabetes and hypertension spiked significantly in relation to the period before the pandemic (20).

#### Topics searched

In Zakar et al. (24) respondents were asked to name the topic they had searched for the most and thus could only give one answer. However, in the other four, the respondents mentioned all the topics that they had searched for.

As mentioned in Table 3 the most searched topics were restrictions in Abdoh (13), Bak et al. (14), the current spread of COVID-19 in Dadaczynski et al. (15), symptoms of COVID-19 in Htay et al. (17) and Zakar et al. (24) and current situation assessments and recommendations in Bak et al. (14). The second most searched topics were the current spread of COVID-19 in Bak et al. (14) Htay et al. (17), Zakar et al. (24), restrictions in Dadaczynski et al. (15) and COVID-19 symptoms in Abdoh (13). The least sought topics were “dealing with psychological stress caused by COVID-19” in Bak et al. (14), Dadaczynski et al. (15), Htay et al. (17), hygiene regulations in Zakar et al. (24) and economic and social consequences of the COVID-19 in Abdoh (13).

**Table 3:** Topics covered by the literature.

Study	Topics								
	COVID-19 symptoms	Restrictions	Current spread of COVID-19	Preventive measures	Transmission routes of COVID-19	Dealing with psychological stress caused by COVID-19	Current situation assessments and recommendations	Hygiene regulations	Economic and social consequences of the COVID-19
Abdoh (13)	95.80%	84.30%	79.40%	74.80%	60.50%	54.60%	54.20%	50.30%	47.70%
Bak et al. (14)	46%	49%	55%	16%	27%	15%	49%	28%	29%
Dadaczynski et al. (15)	71.50%	85.90%	89.60%	45.50%	54.10%	20.70%	77.80%	41%	62.10%
Htay et al. (17)	83.80%	55.70%	91.20%	70.10%	68.70%	39.10%	62.60%	64.50%	54.90%
Zakar et al. (24)	15.10%	1.50%	57.50%	5.20%	5.90%	2.20%	2.70%	1.50%	3.20%

The percentages for Zakar et al. (24) are relatively low compared to the other studies because the percentages were calculated differently.

In Mangono et al. (18), the most searched topics were care seeking for COVID-19, social distancing, online shopping, and COVID-19–specific stimulus packages. In Rovetta and Bhagavathula (22), the top searches were symptoms of COVID-19, followed by face masks and disinfectants. In Zhang et al. (25), the information that was sought out most was about healthy life (90.4%), mental health (56.2%), and information on how to diagnose a disease (70.3%). Zhao and Basnyat (26) which was done in China on a platform called Weibo, was flooded with a search for information related to treatment and condition management, like how to conduct self-quarantine and how to seek offline health care. The results of this study are not diverse because the authors only looked at one search term, “#COVID-19 Patient Seeking Help” and not the entire microblogging platform.

Dadaczynski et al. (15) found gender to be a factor in the topic of information being sought. In the study, as compared to females, males looked more for information regarding the consequences (economic and social) of the COVID-19 pandemic (see Table 3).

### Impact of HISB on vaccine intention

Neely et al. (19) looked at how HISB affects a person’s decision to receive the COVID-19 vaccine. The study found that people who had sought and received their information from credible scientific sources were significantly more likely to be vaccinated against COVID-19. Participants who followed at least two credible sources were 10% more likely to “definitely get vaccinated” than those who did not ascribe to such sources. This number increased to 25% when the number of sources was increased to at least four. In contrast, 30.4% of those who did not follow any credible source said they would not undergo vaccination.

### Digital health literacy

Digital health literacy is the ability to use digital technologies to find, comprehend, and use health information to support health-related decision-making and self-management (10). This includes accessing and evaluating online health information, using digital health tools and resources, and communicating and sharing health information with others.

One widely-used tool for measuring DHL is the Digital Health Literacy Scale (DHLS) (13–15, 23, 24), which consists of a set of items that are designed to assess an individual’s skills in five domains of DHL: access, evaluation, use, communication, and advocacy (10, 28). The scales of the DHLS are: information searching skill, adding self-generated content, competency of evaluating information reliability, and skills in determining information relevancy, as used in Abdoh (13), Bak et al. (14), Dadaczynski et al. (15), and Zakar et al. (24). Vrdelja et al. (23) did not have the item for adding self-generated content. Bak et al. (14) and Dadaczynski et al. (15), had an additional item to access online privacy.

According to Eysenbach et al. (10), one direct effect of DHL is that it affects the choice and use of information sources. There also seems to be a relationship between the DHL and the quality and relevance of the health information that an individual seeks out and uses. This paper looks at each of the DHLS items to explore the areas where participants had difficulties and where there was ease. It also looks at the relationship between the aforementioned items and health information-seeking behavior (HISB). This will help in understanding the HIS behavior of the participants by looking at the effect that DHL has on the HISB of the study group.

The items of the DHLS are looked at individually, reported on, and compared across studies. This is in summary.

#### Information search

In all of the studies, the participants reported it had been easy for them to find information online (13–15, 23, 24). Bak et al. (14) had the highest population percentage at 92.7% for this item. Dadaczynski et al. (15) had a value of 70%, and Vrdelja et al. (23) had a value range of 70.4–94.5%.

#### Determining information relevancy

Difficulties in determining the personal relevancy of information were 32.6% in Abdoh (13), 13.6–17.7% in Vrdelja et al. (23) and 31.5% in Zakar et al. (24). 89.1% of the respondents in Bak et al. (14), 14.4% in Dadaczynski et al. (15), and 69.9% in Zakar et al. (24) believed that the information could be used in everyday decisions.

#### Adding self-generated content

Seventy six and 74.4% of the population in Abdoh (13), 28.9 and 33.9% in Dadaczynski et al. (15); 67.9 and 62.7% in Zakar et al. (24), respectively, found it easy to share their opinions in the form of writing, e.g., social media posts; and to write messages that other people can understand.

#### Evaluating reliability

42.3 and 38.9% of participants in Dadaczynski et al. (15), 64.5 and 53.9% in Zakar et al. (24), respectively, found it challenging to evaluate the reliability of acquired information and to determine whether the information was written with the purpose on eliciting social interest. In Vrdelja et al. (23), 19.1–40.4% of the participants found it difficult to evaluate information reliability.

#### Online privacy

In Bak et al. (14), 74.4% of the respondents found it difficult to decide who could view their post messages and how to protect their privacy. Thirty five percent of the participants in Dadaczynski et al. (15) and 33.5% in Zakar et al. (24) found it difficult to decide and limit who could read their web posts.

### Digital health literacy relationship with HISB

This section seeks to understand the relationships between DHL and information sources. For example, In the case of overall DHL, Bak et al. (14) reported that respondents with sufficient overall DHL used social media significantly less.

Respondents with acceptable DHL in the “information search” subscale used search engines the most in Abdoh (13), Dadaczynski et al. (15), and Vrdelja et al. (23). The second most common source was social media in Abdoh (13), news portals in Dadaczynski et al. (15), and websites of public bodies plus Wikipedia in Vrdelja et al. (23). Those with limited DHL in this subscale used news portals in Abdoh (13), blogs on health topics and support-communities in Dadaczynski et al. (15); and social media, blogs, e-counseling and health portals in Rosário et al. (21) and Vrdelja et al. (23).

Vrdelja et al. (23) also reported that study participants who showed sufficient DHL in information searching considered only up-to-date and verified information and diligently appraised information received.

Respondents with sufficient DHL in the adding self-generated content subscale mostly used search engines in Abdoh (13) and websites of public bodies in Dadaczynski et al. (15). The second most used source for this subscale was social media in Abdoh (13) and Dadaczynski et al. (15). The least used source was news portals in Abdoh (13) and blogs on health topics and support-communities in Dadaczynski et al. (15). Htay et al. (17) reported that sufficient skill in the item of “adding self-generated content” was positively correlated to the use of reliable information sources. The study described credible sources as websites of public institutions, health portals, doctor’s or insurance companies’ websites, and news portals.

Respondents with acceptable DHL in the “Evaluating reliability” subscale used search engines in Abdoh (13), social media in Bak et al. (14) and websites of public bodies in Dadaczynski et al. (15). The least used sources for this subscale were news portals in Abdoh (13) and social media and support communities in Dadaczynski et al. (15) and Rosário et al. (21). In Htay et al. (17), sufficient skills in assessing reliability were positively correlated to the use of reliable information sources. High DHL levels in this subscale were associated with using one specific online source, searching more frequently on health portals, and using Wikipedia and other online encyclopedias as information sources (21). In Vrdelja et al. (23), participants with sufficient DHL on this subscale used websites of public institutions and Wikipedia substantially more frequently than those with limited DHL, who preferred social media, blogs, web counseling, and health portals.

Respondents with acceptable DHL in the “Determining relevance” subscale used search engines in Abdoh (13), websites of public bodies in Dadaczynski et al. (15), the second most used source was social media in Abdoh (13) and search engines in Dadaczynski et al. (15). The least used sources for this domain were news portals in Abdoh (13), social media in Bak et al. (14), and support communities in Dadaczynski et al. (15). In Htay et al. (17), sufficient skills in determining relevancy were positively related to the use of trustworthy information sources. In Vrdelja et al. (24), respondents with sufficient DHL on determining information relevance often used websites of public institutions and rarely used social media, blogs, web counseling services, and health portals compared to those with low DHL.

Studies investigate the relationship between DHL and health information seeking behavior (HISB) among university students during the COVID-19 pandemic (13–15, 17). The studies use different measurements to assess DHL and HISB (13–15, 17, 21, 23). For DHL, some studies use the Digital Health Literacy Instrument (DHLI), which measures the ability to find, understand, appraise, and apply digital health information (21). Other studies use self-reported measures of DHL (13, 15, 23), such as asking participants to rate their ability to understand health information online or their confidence in using digital health tools (17). For HISB, the studies use various measurements, such as frequency and duration of health information seeking, types of sources used for seeking health information, and satisfaction with the information found (13–15, 17).

The results of the studies vary, with some finding a positive association between DHL and HISB, while others finding no significant association (13–15, 17, 21, 23). Some studies also find that certain factors, such as age, gender, and previous experience with online health information, can affect the relationship between DHL and HISB (15, 17). Overall, the studies highlight the importance of promoting DHL among university students and understanding the factors that influence their health information seeking behavior during the pandemic.

#### Factors affecting DHL scores

In Dadaczynski et al. (15) female university students and younger participants showed lower DHL across all subscales. Zakar et al. (24), on the other hand, reported that the overall mean DHL score was higher for females and younger people. In Rosário et al. (21), male students showed substantially higher levels of DHL in the subscales of adding-self generated content and skills of evaluating reliability compared to females (Table 4).

**Table 4:** Quality assessment of included studies according NOS criteria.

References	Selection	Comparability	Outcome	Total
Abdoh (13)	★★★	★★	★★★	8
Bak et al. (14)	★★★★	★★	★★★★	10
Dadaczynski et al. (15)	★★★★	★★	★★★	9
Hsu (16)	★★★	★★	★★★	8
Htay et al. (17)	★★★★	★★	★★★	9
Mangono et al. (18)	★★★★	★★	★★★	9
Neely et al. (19)	★★★★	★★	★★★	9
Rew et al. (20)	★★★	★★	★★★	8
Rosário et al. (21)	★★★★	★★	★★★	9
Rovetta and Bhagavathula (22)	★★★★	★★	★★★	9
Vrdelja et al. (23)	★★★★	★★	★★★	9
Zakar et al. (24)	★★★★	★★	★★★	9
Zhang et al. (25)	★★★	★★	★★★★	9
Zhao and Basnyat (26)	★★★★	★★	★★★★	10
Zhao et al. (27)	★★★★	★★	★★★	9
Component	Description			
Selection				
1. Representativeness of the exposed cohort	The study sample is drawn from a clearly defined population and is representative of the population that it is intended to represent.			
2. Selection of the non-exposed cohort	The selection of the non-exposed cohort should be drawn from the same community as the exposed cohort. It should be established that the selection of the non-exposed cohort is independent of exposure status.			
3. Ascertainment of exposure	The exposure of interest should be clearly defined and the methods for ascertainment should be valid and reliable.			
Outcome				
4. Demonstration that outcome of interest was not present at start of study	The outcome of interest was not present at the start of the study.			
5. Assessment of outcome	The outcome of interest is determined by objective and reliable means, and the follow-up is long enough for outcomes to occur.			
6. Was follow-up long enough for outcomes to occur?	The follow-up period was sufficient for the outcomes of interest to occur.			
Comparability				
7. Comparability of cohorts on the basis of the design or analysis	The cohorts were comparable on the basis of the design or analysis.			
8. Control for any additional factors	There was control for any additional factors.			
9. Assessment of outcome	The outcome of interest is determined by objective and reliable means, and the follow-up is long enough for outcomes to occur.			

*Note that the maximum score for selection is 5, comparability is 2, and outcome is 3. The total score ranges from 0 to 10.

## Discussion

To understand the HISB on the Internet before and after the COVID-19 pandemic, this S.R. paper applied thematic analysis to data from the included studies. Several themes were evaluated, including search strategy, information sources used, motivation for seeking information, information evaluation, and satisfaction. This review also looked at the digital literacy level of the included participants and how the DHL affected the HISB.

Even though there were variations across studies, the most used information sources, in general, were search engines, news portals, and social media (14–17, 19, 23–25). Some variations may be credited to the study methodology. In studies where the sample was university students, the most used sources were search engines, websites of public bodies, or news portals (14–17, 23–25). One major finding is that many individuals rely on the Internet to obtain health information. However, the quality of the information obtained can vary widely, and individuals need to have a good understanding of DHL to effectively evaluate the information they find (21). This includes being able to determine the credibility and reliability of sources and being able to distinguish between factual information and misinformation.

Another finding is that individuals often lack the skills necessary to effectively search for and evaluate online health information. For example, many individuals do not use Boolean logic when conducting their searches or limit the scope of their queries (15). This suggests a need for greater education and training in DHL to ensure that individuals can find and use high-quality health information online.

The review also highlights the impact of the COVID-19 pandemic on online health information-seeking behaviors (19). Several studies found that there was an increase in the amount of health-related information being searched for online during the pandemic and that individuals were more likely to seek out information about COVID-19 specifically (13, 17, 20). This underscores the need for accurate and reliable information about the pandemic to be readily available online.

Information satisfaction among the studies was also positive, with 85.8% in Zakar et al. (24) and 70.8% in Bak et al. (14). Among the studies, Vrdelja et al. (23) and Zakar et al. (24) participants showed an intention to evaluate the credibility of the information. With a good positive percentage of them laying out requirements for information to be regarded as credible. One such requirement was the verification of received information which was mentioned by 68.7% of the participants in Zakar et al. (24) and 99.3% in Vrdelja et al. (23). Despite the good figures, another study, Hsu (16), reported that only 36.4% of its population had sought a doctor’s or professional view on information received. A large portion, 68.4%, sought discussion with friends, and 57.6% conducted an Internet search. Seeking verification from friends and peers was cited by Suarez-Lledo and Alvarez-Galvez (29) as one reason for spreading misinformation. From the review of % in Hsu (16), Vrdelja et al. (23), and Zakar et al. (24) it can be seen that the intention is there but not the action.

In regard to how the HISB had changed from before to during the COVID-19 pandemic, Mangono et al. (18) showed that online searches for all things related to COVID-19 had gone up. Things like COVID-19 news, self-quarantine, protective measures, online shopping, etc. This change would go on to show in topics like COVID-19 symptoms, travel and lifestyle restrictions, current spread of COVID-19, and preventive measures, which were overall the most searched topics (13–15, 17, 24). One major finding is that many individuals rely on the internet to obtain health information (16, 19, 30). However, the quality of the information obtained can vary widely, and it is important for individuals to have a good understanding of DHL in order to effectively evaluate the information they find. This includes being able to determine the credibility and reliability of sources, and being able to distinguish between factual information and misinformation (18, 20).

Also, when looking at the DHL in all subscales, it became clear that good scores in the DHL meant a well-established and effective HSB. A good DHL generally guides the individual toward a more credible source of information, like government websites. For example, Bak et al. (14) reported that respondents with sufficient overall DHL used social media significantly less. Even though social media does not contain 100% false information, its credibility is vastly questionable compared to government or institutional websites.

## Conclusion

This review looked at two main things, the state of the HISB during the COVID-19 pandemic and how DHL affects the HISB. The HISB of the study participants during the pandemic shows that the participants were searching for COVID-19 related information at a high rate. A look at the information sources and the search topics shows that the public was keen to find the correct information. These results suggest that interventions are needed to improve DHL and HISB, such as targeted educational programs or the development of user-friendly online health information resources. Ultimately, improving DHL and HISB can lead to better health outcomes and contribute to mitigating the negative impact of the infodemic during the pandemic.

## Limitations

One limitation of this review was the lack of a study that looked at the HISB both during and after the COVID-19 pandemic. This meant that no during-after comparison could be made.

Another limitation, though minor, was the variability in the study scopes and how the results were presented. This means that themes, e.g., the relationship between DHL and HISB, were not reported in all studies. Even though this S.R. paper contains 15 studies, not all of them are included in the analysis of each theme explored in this paper; this was not a huge challenge because the studies were enough for the theme to be studied.

## Author contributions

HA wrote the entire review.

## Funding

This work was supported by the Deanship of Scientific Research, Qassim University.

## Conflict of interest

The author declares that the research was conducted in the absence of any commercial or financial relationships that could be construed as a potential conflict of interest.

## Publisher’s note

All claims expressed in this article are solely those of the authors and do not necessarily represent those of their affiliated organizations, or those of the publisher, the editors and the reviewers. Any product that may be evaluated in this article, or claim that may be made by its manufacturer, is not guaranteed or endorsed by the publisher.
